# Polyethylene Microplastic-Induced Changes in Soil Properties Mediate Nutrient Accumulation and Growth of *Amaranthus tricolor* L.

**DOI:** 10.3390/plants15111720

**Published:** 2026-06-02

**Authors:** Luqing Yang, Mengyang Wang, Jie Cheng, Jianghu Long, Lun Wang, Jiaqi Liu, Wen Zhai, Junqi Liu, Lisheng Feng, Yang Luo

**Affiliations:** School of Geography and Resources, Guizhou Education University, Guiyang 550018, China; luqing39@outlook.com (L.Y.); mywang2005@outlook.com (M.W.); chengjie200504@outlook.com (J.C.);

**Keywords:** polyethylene microplastic, *Amaranthus tricolor* L., plant physiology, soil physical and chemical properties, soil bacterial community

## Abstract

The impact of microplastic pollution on soil functions and the ecological toxicity to crops is a hotspot in agronomy and environmental science. In this study, a pot experiment was conducted to examine the effects of polyethylene microplastics (PE-MPs) at concentrations of 0.25%, 0.5%, 2.5%, and 5% on the growth, physiological indicators, soil physical and chemical properties, and soil bacterial community diversity of *Amaranthus tricolor* L. The results showed that adding PE-MPs inhibited the growth of *A. tricolor* L. stems and leaves, and as the dosage increased, the aboveground fresh weight decreased by 13.5% to 60.7%. At the same time, the chlorophyll content in *A. tricolor* L. leaves decreased, whereas the malondialdehyde (MDA) content increased by up to 11.8%. When the added dosage of PE-MPs was ≥0.5%, soil porosity and available phosphorus (AP) content significantly decreased, resulting in a significant reduction of 22.1% to 31.3% in the phosphorus content of the aboveground parts of *A. tricolor* L. compared with controls (CK). High-throughput sequencing results indicated that adding PE-MPs could reduce the Shannon index of the soil bacterial community and increase the Simpson index, suggesting a decrease in diversity. The addition of PE-MPs also altered the structure of the soil bacterial community, increasing the relative abundance of the Acidobacteriota, while the abundance of the Planctomycetota significantly decreased. This study provides a numerical and theoretical basis for evaluating the impact of microplastics on vegetable production and soil ecological environment effects.

## 1. Introduction

Plastic mulch films, greenhouse films, and irrigation pipes are widely used in agriculture to enhance crop yields, conserve water, and protect plants from pests, diseases, and adverse weather conditions. These plastic products are difficult to recycle and, when retained in the soil, gradually decompose into microplastic particles with diameters less than 5 mm [1,2]. Previous studies have shown that once microplastics enter the soil, their small size, hydrophobicity, and strong adsorption capacity alter soil physicochemical, abiotic, and biological properties. For example, they affect soil nutrient cycling, modify soil physical and chemical characteristics, and influence microbial community composition [3,4,5], thus impacting plant growth. Polyethylene microplastics (PE-MPs) are among the most prevalent types of microplastics in agricultural soils. According to Hu et al. [6], polyethylene (PE) accounts for 18% of microplastics in farmland soils in China. PE mulch films are relatively thin, prone to fragmentation, and have a low recovery rate. Their long-term accumulation can lead to severe microplastic contamination in farmland soils [7], adversely affecting crop growth and food security. Consequently, investigating the potential threats posed by PE-MPs to soil ecological environments and plant growth has become a research focus in agronomy and environmental science.

The concentration of PE-MPs in the medium is a key factor determining their ecotoxicity. Zhang et al. [8] used ryegrass as the test plant and observed that PE-MPs promoted seed germination and seedling elongation at added concentrations of 100 mg/L and 200 mg/L, whereas a clear inhibitory effect was observed at a concentration of 1000 mg/L. Cai et al. [9] reported that lettuce growth was not significantly affected at a PE-MP concentration of 0.05%. In contrast, when the concentration was increased to 0.1%, a significant reduction in dissolved organic carbon of root exudates was observed, accompanied by a decrease in biomass. Furthermore, differences in physiological structure and genetic characteristics cause specific plant types to exhibit varying toxicological responses when exposed to the same microplastic-contaminated soil environment. For instance, Wang et al. [10] observed that at a PE-MP concentration of 500 mg/L in soil, mung bean growth was promoted, whereas soybean root length was significantly reduced by 50.1%. The findings of Sahasa et al. [11] confirmed that the inhibitory effect of PE-MPs on the growth of black gram was greater than that on tomato across different microplastic concentrations. Therefore, studies on the influence of microplastic concentration and plant type have yielded no consistent pattern regarding the toxic effects of soil PE-MP pollution on plants.

*Amaranthus tricolor* L. is an annual vegetable in the family *Amaranthaceae*. This vegetable is highly nutritious, containing protein, vitamin C, and a variety of essential minerals, including calcium, iron, magnesium, phosphorus, potassium, and sodium [12]. In addition to its use as a food crop, *A. tricolor* L. has medicinal value, as it helps alleviate dysentery symptoms, promote cell regeneration, and accelerate recovery [13,14]. In the agricultural production of *A. tricolor* L., plastic mulch coverings are commonly used, as in most vegetable cultivation practices, to suppress weed growth and enhance soil warming and moisture retention [15]. However, the risks associated with microplastic pollution warrant further attention. In summary, PE-MPs were thoroughly mixed with soil at specific ratios, followed by a pot experiment in which plant morphological, growth, and physiological indicators, as well as the response of *A. tricolor* L. to PE-MP stress, were investigated. Combined with analyses of soil physicochemical properties and bacterial community data, the underlying mechanisms were preliminarily elucidated. This study aims to provide a theoretical basis for clarifying the ecological risks posed by PE-MPs in soil to promote the safe cultivation of *A. tricolor* L.

## 2. Results

### 2.1. A. tricolor L. Seed Germination Status

**Figure 1:**
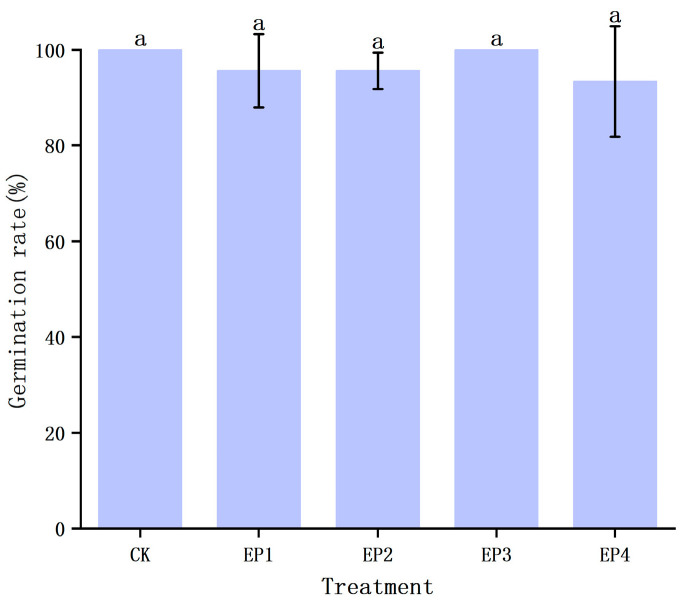
Germination rate of *A. tricolor* L. seeds under specific treatments.

The germination of *A. tricolor* L. seeds under different treatments is shown in Figure 1. The germination rate was 100% in control (CK, i.e., no PE-MPs) and in the treatment with 2.5% PE-MPs (EP3). At added PE-MP levels of 0.25% and 0.5% (EP1 and EP2), the germination rate was 95.56% for both treatments. When the added PE-MPs concentration reached 5% (EP4), the germination rate was 93.33%. The results of the variance analysis indicated no significant differences among treatments (*p* > 0.05).

### 2.2. A. tricolor L. Growth Status

**Figure 2:**
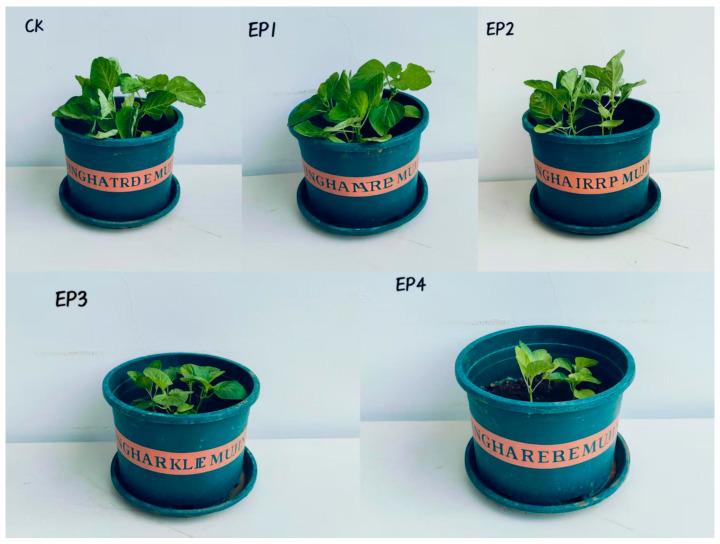
Growth photos of *A. tricolor* L.

The growth photos of *A. tricolor* L. plants under different treatments at harvest is shown in Figure 2. Plants treated with PE-MPs at addition rates of 0.5–5% (EP2–EP4) were sparse and stunted, with few small leaves and some yellowing. As shown in Table 1, after adding PE-MPs at mass ratios ranging from 0.25% to 5% (EP1 to EP4), the plant height, leaf width, and leaf length of *A. tricolor* L. were significantly reduced by 22.3–52.3%, 14.1–35.3%, and 9.2–32.1%, respectively, compared with the CK (*p* < 0.05). The greatest reductions across all three parameters were observed in the EP4 treatment group. Leaf fresh weight of *A. tricolor* L. decreased with increasing PE-MP application rates. Compared with the CK, the reductions were 12.5% (EP1), 28.0% (EP2), 44.2% (EP3), and 60.9% (EP4), respectively. Differences among all treatment groups were statistically significant (*p* < 0.05). Stem fresh weight of *A. tricolor* L. decreased with increasing PE-MP application rates, with reductions ranging from 17.4% to 60.2%. All treatments showed significant differences compared with the CK; however, no significant differences were observed between EP1 and EP2 or between EP3 and EP4. In this study, the aboveground fresh weight of *A. tricolor* L. ranged from 4.66 to 11.87 g per pot, with a decreasing trend as PE application rates increased. Differences among all treatments were statistically significant (*p* < 0.05). Compared with the CK, the aboveground fresh weight of *A. tricolor* L. decreased by 13.5%, 27.0%, 45.41%, and 60.7% after adding 0.25%, 0.5%, 2.5%, and 5% PE-MPs to the soil, respectively (*p* < 0.05).

### 2.3. Chlorophyll and Malondialdehyde (MDA) Content in A. tricolor L. Leaves

As shown in Table 2, no significant differences in chlorophyll a content were observed between the CK and 0.25% and 0.5% PE-MP treatments. However, chlorophyll a content was significantly reduced by 12.4% and 18.6% in the 2.5% and 5% PE-MP treatments, respectively (*p* < 0.05). The chlorophyll b content in *A. tricolor* L. leaves decreased with increasing PE-MPs application rates, with reductions ranging from 20.0% to 45.0%. The total chlorophyll content in *A. tricolor* L. leaves showed no significant change at PE-MPs addition levels of 0.25% and 0.5%, but was significantly reduced by 15.4% and 23.1% at addition levels of 2.5% (EP3) and 5% (EP4), respectively, compared with the CK (*p* < 0.05). No significant difference was observed between EP3 and EP4. In this study, the MDA content in *A. tricolor* L. leaves ranged from 25.93 to 29.41 nmol/g. The order of values across treatment groups was EP4 > EP1 > EP3 > EP2 > CK. Compared with the CK treatment, the MDA content was significantly increased by 10.9% in the EP1 group and by 11.8% in the EP4 group (*p* < 0.05).

### 2.4. Nutrient Contents of A. tricolor L.

The main nutrient contents in the aboveground parts of *A. tricolor* L. were measured, and the results are shown in Table 3. The nitrogen content in the aboveground parts ranged from 36.12 to 39.42 g/kg, and the potassium content ranged from 16.96 to 17.95 g/kg. No significant differences were observed for either of these two indicators among treatments (*p* > 0.05). The phosphorus content in the aboveground parts of *A. tricolor* L. in the CK group was 1.63 g/kg. After adding PE-MPs, it decreased significantly to a range of 1.12 to 1.31 g/kg (*p* < 0.05), representing a reduction of 19.6% to 31.3%. However, no significant differences were observed among the four PE-MPs treatments.

### 2.5. Basic Physicochemical Properties of the Soil

The main physicochemical properties of the soil under individual treatments are shown in Table 4. Notably, no significant differences were found in soil pH, bulk density (BD), organic matter (OM), or available nitrogen (AN) contents across all treatment groups. When the PE-MP application rate reached or exceeded 0.5%, soil total porosity (TP) was reduced significantly from 56.9% in the CK group to 52.2% (EP2), 52.6% (EP3), and 51.2% (EP4), respectively. Compared with CK, soil available phosphorus (AP) content was significantly reduced by 14.1%, 17.9%, and 12.2% when PE-MPs were added at mass ratios of 0.5% (EP2), 2.5% (EP3), and 5% (EP4), respectively (*p* < 0.05). However, no significant differences were observed among the three treatments. The order of soil available potassium (AK) content was EP3 > EP2 > EP1 > CK > EP4, and no significant differences were observed between the four PE-MP treatment groups and the CK (*p* > 0.05).

### 2.6. Correlation Analysis Between Soil Physicochemical Properties and A. tricolor L. Growth Indicators

**Figure 3:**
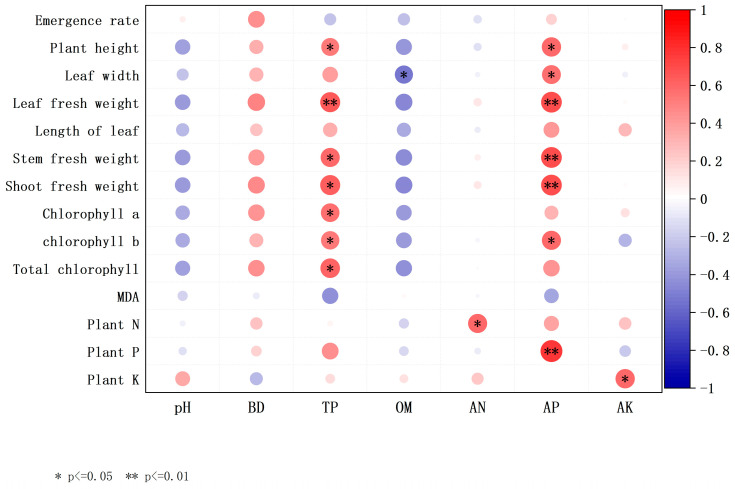
Correlation heatmap of soil physicochemical properties and *A. tricolor* L. growth indicators.

Pearson correlation analysis was conducted between soil physicochemical parameters and various growth and physiological indicators of *A. tricolor* L. The results are shown in Figure 3. Soil pH and BD showed no significant correlations with the growth or physiological indicators of *A. tricolor* L. Soil TP showed a significant positive correlation with plant height, stem fresh weight, and aboveground fresh weight, as well as the chlorophyll a, chlorophyll b, and total chlorophyll contents of *A. tricolor* L. (*p* < 0.05) and a highly significant positive correlation with leaf fresh weight (*p* < 0.01). Soil OM was significantly negatively correlated only with leaf width of *A. tricolor* L. (*p* < 0.05) and showed no significant correlations with other indicators. Soil AN was significantly positively correlated only with the nitrogen content of *A. tricolor* L. Soil AP was significantly positively correlated with plant height, leaf width, and the chlorophyll b content of *A. tricolor* L. (*p* < 0.05) and highly significantly positively correlated with leaf fresh weight, stem fresh weight, aboveground fresh weight, and the phosphorus content of *A. tricolor* L. (*p* < 0.01). Soil AK was significantly positively correlated only with potassium content of *A. tricolor* L. and showed no significant correlations with other growth or physiological indicators of *A. tricolor* L. (*p* < 0.05).

### 2.7. Soil Bacterial Community

Table 5 presents the diversity indices of soil bacterial communities under various treatment conditions. Notably, the Shannon indices of soil bacterial communities in the PE-MP treatment groups ranged from 6.36 to 6.51, lower than the CK value of 6.57. Except for the EP1 treatment, the differences from the CK were statistically significant (*p* < 0.05). The Ace index of the soil bacterial community ranged from 3899.18 to 4102.84; no significant differences were found across all treatment groups. Regarding the Simpson index, all PE-MPs treatment groups exhibited higher values than the CK group, with significant differences observed between the CK and the EP3 and EP4 treatments.

**Figure 4:**
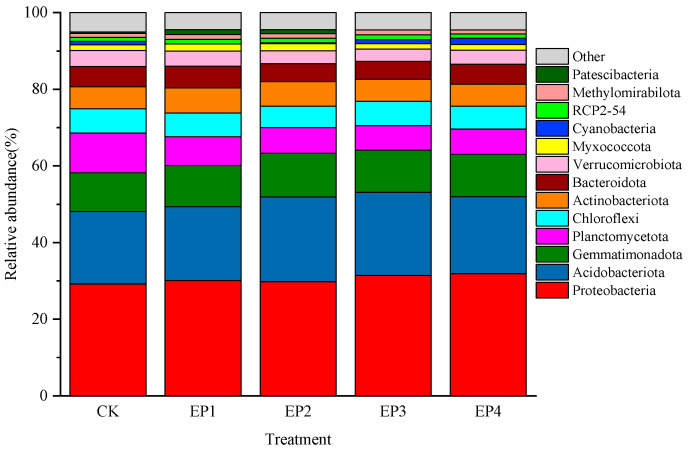
Relative abundance of soil bacteria at the phylum level under PE-MP treatments.

The phylum-level classification of soil bacterial communities under different treatment conditions is shown in Figure 4. Notably, when excluding the Other group, the soil bacteria were mainly distributed across 13 phyla. Among them, the highest relative abundance was Proteobacteria (ranging from 29.18% to 31.89%), with no significant differences among treatments. Next was Acidobacteriota (ranging from 18.95% to 22.12%); all PE-MPs addition treatment groups were higher than the control group, among which the differences between EP2 and EP3 and the control reached significant levels. The third highest relative abundance was Gemmatimonadota (ranging from 10.13% to 11.42%), with no significant differences among treatments. Among the other bacterial phyla, the relative abundance of Planctomycetota in the four PE-MP treatment groups showed a significant decreasing trend compared with the CK treatment.

## 3. Discussion

### 3.1. Mechanism by Which PE-MPs Affect A. tricolor L. Growth

Plant growth refers to an increase in plant volume or mass, which is closely associated with cell differentiation, proliferation, and physiological activities. When the growing environment deviates from the optimal state, these mechanisms may be altered, impairing plant growth and development. Previous studies have shown that microplastics can accumulate in seed coat pores, thus affecting the seed’s water uptake capacity and delaying germination [16]. In the present study, adding PE-MPs at mass ratios ranging from 0.25% to 5% did not significantly affect the germination of *A. tricolor* L. seeds. This finding may be attributable to the fact that, with increasing exposure time to PE-MPs, the seeds had already absorbed sufficient water. Under favorable temperature conditions, the intrinsic nutrients within the seeds may have provided adequate energy and material support for embryo growth and development [17].

As a leafy vegetable primarily consumed for its vegetative parts, the economic yield and nutritional value of *A. tricolor* L. are mainly concentrated in its aboveground portions. In cultivation practice, the total aboveground biomass, including stems and leaves, constitutes the final harvested product and directly determines the economic return of *A. tricolor* L. This study showed that after adding PE-MPs, plant height, leaf width, leaf length, and fresh weight of *A. tricolor* L. were significantly lower than those under the CK treatment. These findings are consistent with the conclusions of Liu et al. [18], who investigated the toxic effects of microplastics on tomato seedlings. Moreover, the inhibitory effect became more pronounced as the PE-MP mass fraction increased. Considering the limited capacity of plants to directly absorb microplastics from soil, the primary cause of this phenomenon is likely the alteration of the soil environment induced by microplastics [19,20,21], in turn increasing stress on *A. tricolor* L. growth. In this study, adding PE-MPs, particularly at mass fractions of 0.5% or higher, resulted in reductions in soil porosity and AP content, both of which were mostly significantly or highly significantly positively correlated with *A. tricolor* L. growth parameters. Coupled with the decrease in soil bacterial community diversity and the relative abundance of beneficial bacterial phyla, these changes further adversely affected soil nutrient cycling and the uptake of nutrients by *A. tricolor* L. Consequently, the aboveground phosphorus content of *A. tricolor* L. in the MP-amended treatment groups was significantly lower than that in the control group, but no significant difference was observed among the different MP dosage treatment groups. Possibly because phosphorus accumulation in plants depends not only on soil available phosphorus supply but also on the expression of phosphorus transporters and the plant’s phosphorus uptake strategy. Under microplastic stress, when soil AP declines to a certain level, plants may compensate for the reduced supply by adjusting their phosphorus uptake or use efficiency, which could explain why aboveground phosphorus content did not decrease further among the treatment groups [22]. In addition, the chlorophyll content of *A. tricolor* L. decreased, inhibiting photosynthesis and exacerbating oxidative damage to cell membranes [23], leading to excessive production of reactive oxygen species and marked accumulation of MDA, a product of membrane lipid peroxidation [24], thus impairing plant growth and development.

### 3.2. Mechanism by Which PE-MPs Affect Soil Properties

In this study, no change in soil BD was observed under PE-MPs treatments, whereas soil TP decreased significantly. This finding may be attributable to the low microplastic density. However, their incorporation into the soil did not markedly alter the solid-phase mass per unit volume; they may have reduced soil porosity by filling existing pore spaces, disrupting pore connectivity, and altering contact patterns among soil aggregates [19,25]. Further, we observed that adding PE-MPs reduced soil AP content, consistent with the findings of Li et al. [26]. Possible reasons are as follows. First, microplastics can alter the diversity and composition of soil bacterial communities, which may further affect the functions of microbes involved in organic phosphorus mineralization and inorganic phosphorus solubilization, thereby changing the release of available phosphorus in soil. Second, microplastics can reduce phosphatase activity by changing substrate accessibility, adsorbing enzyme molecules, or affecting enzyme-producing microbial communities, leading to a decline in the conversion efficiency of organic phosphorus to inorganic phosphorus and indirectly lowering soil available phosphorus levels [27]. Third, microplastics themselves or the complexes they form with soil minerals may increase the number of adsorption sites for phosphorus, thus altering the partitioning of phosphorus between solid and liquid phases. In addition, additives released during microplastic degradation, such as plasticizers and flame retardants, may compete with phosphate ions for adsorption sites or form complexes with ions like iron and aluminum, further interfering with phosphorus availability [28,29]. In summary, the impact of microplastics on soil phosphorus availability is not through a single pathway but results from multiple processes, including changes in microbial communities, enzyme activities, and nutrient dynamics. The mechanisms may vary with microplastic type, particle size, and concentration, and further research is needed.

Bacteria can respond rapidly to soil disturbance or contamination, rendering them sensitive indicators of the real-time health status of soil [30]. In general, higher diversity and richness indices of bacterial communities indicate more complex community structures and enhanced stability. In this study, compared with the CK treatment, the Shannon index of the soil bacterial community in the 5% PE-MP treatment decreased significantly, whereas the Simpson index increased significantly, indicating a reduction in bacterial community diversity [31,32]. Notably, when PE-MPs in the soil reach a certain concentration, they can degrade the soil environment, thus inhibiting bacterial growth and reproduction and ultimately affecting the overall stability of the soil ecosystem. Under the disturbance of microplastics, shifts in soil bacterial community structure generally indicate corresponding changes in ecosystem functions [33]. This study showed that adding PE-MPs at varying concentrations significantly reduced the relative abundance of Planctomycetota in the soil. At added PE-MP levels of 0.5% (EP2) and 2.5% (EP3), the relative abundance of Acidobacteriota increased significantly. Planctomycetota is an important functional bacterial group involved in carbon cycling in soils. Its mutualistic interactions with the soil include the degradation of complex modified polysaccharides produced by photosynthetic organisms [34]. In addition, it is a versatile phylum capable of degrading cellulose and various non-cellulosic biopolymers [35], playing a crucial role in maintaining soil OM turnover and structural stability. Furthermore, Planctomycetota also represent an important phosphorus-cycling functional bacterial group, capable of secreting phosphatases, thereby increasing phosphorus availability [36]. Acidobacteriota are oligotrophic microorganisms characterized by low metabolic rates, high carbon substrate utilization efficiency, and high tolerance to low pH and low-nutrient environments. Meanwhile, some members possess the ability to secrete extracellular polymeric substances to resist exogenous toxicant stress, thereby gaining a competitive advantage under stress conditions [37,38,39]. Therefore, the changes in soil bacterial composition at the phylum level induced by PE-MPs may indicate community dysbiosis, posing a potential threat to the ecological functions of soil microorganisms.

Although this study combined analyses of plant physiological responses, soil properties, and microbial communities and made some progress in elucidating the ecotoxicological mechanisms of PE-MPs on *A. tricolor* L., the following limitations remain. First, the toxicity of microplastics largely depends on particle morphology, size distribution, surface properties, and chemical additives. Future studies should characterize the microplastics more comprehensively to enhance the robustness of the conclusions. Second, in plant pot toxicology studies, three replicates per treatment is a common practice. However, for microbial community analysis, diversity index calculation, and multivariate correlation analysis, this sample size is too small and provides limited statistical power, which may affect the reliability of the results. Future studies should use more reasonable numbers of replicates depending on the analytical endpoints. Third, due to the limited size of the pots, only 15 seeds were sown per pot. This small sample size in the germination test may amplify random errors and thus affect the representativeness and reproducibility of the results. Future studies should increase the number of seeds to obtain more robust conclusions. Fourth, although fresh weight is the reference standard for *A. tricolor* L. in both consumption and commercial distribution, it is susceptible to interference from plant water status. Future studies should include dry weight measurements to more accurately assess changes in biomass accumulation.

## 4. Materials and Methods

### 4.1. Test Materials

The PE-MPs used in this experiment were purchased from Feihong Plastic Chemical Company, Zhangmutou Town, Dongguan City, China, with a particle size of 300 mesh (48 μm). According to the manufacturer’s Certificate of Analysis for the batch used, the polymer purity of both types exceeded 99%, and the particles were spherical in shape.

The *A. tricolor* L. seeds used in this experiment were purchased from Mu Yang Nian Guo Duo Flowers Co., Ltd. (Shuyang, China). Before use, the seeds were rinsed with tap and distilled water, and those floating on the water surface were discarded. The remaining seeds were sterilized by immersion in 3% H_2_O_2_ (*v*/*v*) for 30 min, rinsed repeatedly with distilled water to remove residual H_2_O_2_ from the seed surface, and blotted dry with filter paper.

The soil used in this experiment was collected from sloping land around Guizhou Education University. The soil type was calcareous soil, with a BD of 1.08 g/cm^3^, pH of 7.28, OM content of 37.97 g/kg, AN content of 133.07 mg/kg, AP content of 12.10 mg/kg, and AK content of 137.08 mg/kg.

### 4.2. Experimental Design

The pot experiment was conducted at Guizhou Education University in Wudang District, Guiyang City. To keep the environmental conditions close to the natural state and thereby enhance the applicability of the results to real agricultural scenarios, the experimental site was a semi-open, naturally ventilated greenhouse with all sides fully open, and no artificial control of temperature or light was applied. According to local meteorological data, the daily average temperature during the experiment ranged from 18 °C to 30 °C. This study included five treatments: ① Control: CK, no added PE-MPs; ② EP1: PE-MPs added at a mass ratio of 0.25%; ③ EP2: PE-MPs added at a mass ratio of 0.5%; ④ EP3: PE-MPs added at a mass ratio of 2.5%; ⑤ EP4: PE-MPs added at a mass ratio of 5%. The highest concentration of microplastics used in this study reached 5% (*w*/*w*). This was based primarily on two considerations. First, to simulate extreme contamination scenarios that may occur in agricultural production. Studies have reported that the mass fraction of MPs in soil ranges from 0.03% to 6.75% [40]. Second, existing plant toxicology studies indicate that high-dose exposure helps reveal the potential ecotoxicological effects of pollutants and provides a basis for risk screening [41]. Each treatment was performed in triplicate. PE-MPs were mixed with soil at the designated ratios and placed into pots lined with gauze. The mixtures were stirred continuously until homogeneous, with each pot containing 1.8 kg. The experiment commenced after a 30-day equilibration period. To avoid interference with the experimental results, no fertilizer was applied during the entire pot experiment period.

*A. tricolor* L. seeds that were plump and uniform in maturity were selected, cleaned, and sown in each pot at a rate of 15 seeds per pot. Twenty days after sowing, the seedlings were thinned to four plants of comparable growth per pot. To minimize the influence of the outdoor environment during the experiment, the positions of the pots were randomly rearranged every three days. Water was applied regularly to maintain soil moisture suitable for seed germination (daily irrigation using the gravimetric method ensured soil moisture remained at approximately 65% of field capacity). The plants were harvested 70 days after seed sowing. The aboveground parts of the *A. tricolor* L. plants were rinsed with deionized water, blotted dry with sterilized filter paper, and then used to measure morphological parameters and fresh weight. A portion of the fresh samples was retained for the determination of plant physiological characteristics. The remaining samples were oven-dried at 105 °C for 20 min to deactivate enzymes, then dried at 70 °C to a constant weight for nutrient analysis. Meanwhile, rhizosphere soil samples were collected, and debris was removed. Each sample was divided into two portions: one portion was air-dried indoors and passed through 2 mm and 0.15 mm sieves to determine soil physicochemical properties; the other was stored in a −80 °C freezer for analysis of soil bacterial community indices.

### 4.3. Indicator Measurement and Methodology

#### 4.3.1. Determination of Plant Indicators

Plant height, leaf width, and leaf length of *A. tricolor* L. were measured using a ruler. Fresh weights of stems and leaves were determined using an analytical balance with a precision of 0.0001 g. Chlorophyll was extracted with 95% ethanol, and absorbance was measured at 649 nm (chlorophyll b) and 665 nm (chlorophyll a) to calculate their respective contents [42]. The MDA content was determined using the thiobarbituric acid colorimetric method [43].

#### 4.3.2. Determination of Soil Indicators

Soil BD was determined using the cutting ring method. Soil pH was measured with a glass-electrode pH meter. Soil OM was determined using the potassium dichromate oxidation method [44]. Soil AN was measured using the alkaline diffusion method [45]. Soil AP was determined using the sodium bicarbonate–molybdenum antimony colorimetric method. Soil AK was extracted with acetic acid and analyzed using a flame photometer [46]. Soil bacterial community analysis was conducted by Sangon Biotech (Shanghai, China). Soil DNA was extracted using the E.Z.N.A.™ Mag-Bind Soil DNA Kit (Omega Bio-tek, Inc., Norcross, GA, USA), and high-throughput sequencing was performed on the Illumina MiSeq platform [47].

### 4.4. Data Processing

Data processing and calculations were performed using Excel 2019, and graphs were generated using Origin 2022. One-way analysis of variance was conducted using SPSS 26, and differences between groups were compared using the least significant difference method. Pearson’s correlation analysis was also performed using SPSS 26. The Ace, Shannon, and Simpson indices of soil bacteria were calculated using Mothur software (version 1.48.0).

## 5. Conclusions

The addition of PE-MPs did not affect the emergence of *A. tricolor* L. seeds; however, it significantly inhibited stem and leaf growth, with the degree of inhibition increasing with application rate. High mass fractions (2.5% and 5%) of PE-MPs significantly reduced chlorophyll content in *A. tricolor* L., thus affecting its photosynthesis. The addition of PE-MPs inhibited the uptake of phosphorus by *A. tricolor* L. and induced oxidative damage to the cell membrane. PE-MPs had no significant effects on soil pH, BD, OM, or AN. However, within the application range of 0.25% to 5%, PE-MPs significantly inhibited soil porosity and AP content. In addition, PE-MPs reduced the diversity of the soil bacterial community and significantly decreased the relative abundance of Planctomycetota. When PE-MPs were added at concentrations of 0.5% and 2.5%, the relative abundance of Acidobacteriota increased significantly. Therefore, attention should be paid to the potential risks associated with the accumulation of PE-MPs in *A. tricolor* L. production. In the future, the negative impacts of microplastics on crop growth and soil health could be mitigated by controlling the residual amount of plastic mulch and using degradable mulching materials.

## Figures and Tables

**Table 1:** Growth parameters of *A. tricolor* L.

Treatment	Plant Height (cm)	Leaf Width (cm)	Leaf Length (cm)	Leaf Fresh Weight (g/pot)	Stem Fresh Weight (g/pot)	Shoot Fresh Weight (g/pot)
CK	9.58 ± 0.29 a	5.67 ± 0.42 a	6.53 ± 0.42 a	9.51 ± 1.29 a	2.36 ± 0.23 a	11.87 ± 1.49 a
EP1	6.75 ± 0.35 c	4.10 ± 0.10 c	5.03 ± 0.15 c	8.32 ± 0.30 b	1.95 ± 0.22 b	10.27 ± 0.40 b
EP2	7.40 ± 0.31 b	4.87 ± 0.38 b	5.93 ± 0.25 b	6.85 ± 0.22 c	1.81 ± 0.08 b	8.66 ± 0.15 c
EP3	6.26 ± 0.20 c	3.77 ± 0.15 c	5.30 ± 0.46 c	5.31 ± 0.20 d	1.17 ± 0.10 c	6.48 ± 0.23 d
EP4	4.57 ± 0.41 d	3.67 ± 0.40 c	4.43 ± 0.23 d	3.72 ± 0.16 e	0.94 ± 0.13 c	4.66 ± 0.29 e

Note: Data in the table are presented as mean ± standard deviation. Different lowercase letters within the same column indicate significant differences at the *p* < 0.05 level.

**Table 2:** Chlorophyll and MDA content in *A. tricolor* L. leaves under different treatments.

Treatment	Chlorophyll a (mg/g)	Chlorophyll b (mg/g)	Total Chlorophyll (mg/g)	MDA (nmol/g)
CK	0.97 ± 0.03 a	0.20 ± 0.01 a	1.17 ± 0.03 a	25.93 ±2.11 b
EP1	0.95 ± 0.07 ab	0.16 ± 0.03 b	1.10 ± 0.06 a	29.09 ±2.42 a
EP2	0.92 ± 0.02 ab	0.15 ± 0.03 bc	1.07 ± 0.05 ab	28.46 ±1.13 ab
EP3	0.85 ± 0.04 bc	0.14 ± 0.01 bc	0.99 ± 0.05 bc	28.48 ±1.37 ab
EP4	0.79 ± 0.09 c	0.11 ± 0.01 c	0.90 ± 0.08 c	29.41 ± 1.00 a

Note: Data in the table are presented as mean ± standard deviation. Different lowercase letters within the same column indicate significant differences at the *p* < 0.05 level.

**Table 3:** Nutrient contents in the aboveground parts of *A. tricolor* L. under different treatments.

Treatment	N (g/kg)	P (g/kg)	K (g/kg)
CK	37.15 ± 4.14 a	1.63 ± 0.07 a	17.15 ± 0.69 a
EP1	39.42 ± 0.78 a	1.31 ± 0.13 b	17.43 ± 1.38 a
EP2	36.12 ± 2.47 a	1.12 ± 0.21 b	17.95 ± 1.59 a
EP3	36.89 ± 1.44 a	1.26 ± 0.12 b	17.30 ± 1.10 a
EP4	36.92 ± 1.45 a	1.27 ± 0.05 b	16.96 ± 0.83 a

Note: Data in the table are presented as mean ± standard deviation. Different lowercase letters within the same column indicate significant differences at the *p* < 0.05 level.

**Table 4:** Main physicochemical properties of the soil under specific treatments.

Treatment	pH	BD (g/cm^3^)	TP (%)	OM (g/kg)	AN (mg/kg)	AP (mg/kg)	AK (mg/kg)
CK	7.56 ± 0.06 a	1.09 ± 0.08 a	56.90 ± 2.39 a	38.49 ± 1.36 a	141.91 ± 8.05 a	17.87 ± 0.40 a	127.57 ± 5.35 ab
EP1	7.61 ± 0.05 a	1.10 ± 0.07 a	56.25 ± 3.04 ab	38.44 ± 1.94 a	145.85 ± 4.30 a	16.44 ± 0.81 ab	127.64 ± 7.15 ab
EP2	7.62 ± 0.16 a	1.07 ± 0.02 a	52.23 ± 0.69 c	38.12 ± 1.14 a	143.24 ± 9.38 a	15.35 ± 1.18 bc	134.19 ± 5.26 ab
EP3	7.67 ± 0.13 a	1.03 ± 0.04 a	52.58 ± 1.12 bc	39.75 ± 2.22 a	143.98 ± 1.40 a	14.68 ± 0.60 c	135.14 ± 3.48 a
EP4	7.66 ± 0.12 a	1.06 ± 0.05 a	51.16 ± 2.61 c	40.91 ± 2.23 a	142.74 ± 4.82 a	15.69 ± 0.74 bc	124.80 ± 3.70 b

Note: Data in the table are presented as mean ± standard deviation. Different lowercase letters within the same column indicate significant differences at the *p* < 0.05 level.

**Table 5:** Diversity indices of soil bacterial community structure under PE-MP treatments.

Treatment	Shannon	Ace	Simpson
CK	6.57 ± 0.03 a	4102.84 ± 99.46 a	0.0058 ± 0.0007 b
EP1	6.51 ± 0.07 ab	4098.30 ± 142.90 a	0.0067 ± 0.0013 ab
EP2	6.43 ± 0.03 bc	4020.26 ± 263.05 a	0.0073 ± 0.0001 ab
EP3	6.41 ± 0.07 bc	3975.86 ± 140.16 a	0.0080 ± 0.0009 a
EP4	6.36 ± 0.10 c	3899.18 ± 156.66 a	0.0084 ± 0.0018 a

Note: Data in the table are presented as mean ± standard deviation. Different lowercase letters within the same column indicate significant differences at the *p* < 0.05 level.

## Data Availability

All data necessary to replicate this study’s results are included in this published article. The raw data files of soil bacterial sequencing are available in the NCBI repository under accession number PRJNA1455141 (https://www.ncbi.nlm.nih.gov/sra (accessed on 18 April 2026)).

## Abbreviations

The following abbreviations are used in this manuscript:

AN	Available nitrogen
AP	Available phosphorus
AK	Available potassium
OM	Organic matter
MDA	Malondialdehyde
BD	Bulk Density
TP	Total Porosity
PE-MPs	Polyethylene Microplastics

## References

[1] Nguyen M.K., Rakib M.R.J., Lin C., Hung N.T.Q., Le V.G., Nguyen H.L., Malafaia G., Idris A.M. (2023). A comprehensive review on ecological effects of microplastic pollution: An interaction with pollutants in the ecosystems and future perspectives. Trends Anal. Chem..

[2] Hoang V.H., Nguyen M.K., Hoang T.D., Ha M.C., Huyen N.T.T., Bui V.K.H., Pham M.T., Nguyen C.M., Chang S.W., Nguyen D.D. (2024). Sources, environmental fate, and impacts of microplastic contamination in agricultural soils: A comprehensive review. Sci. Total Environ..

[3] de Souza Machado A.A., Lau C.W., Till J., Kloas W., Lehmann A., Becker R., Rillig M.C. (2018). Impacts of microplastics on the soil biophysical environment. Environ. Sci. Technol..

[4] Zeng R.B., Zi R.Y., Feng Y.Y., Zhou N.A., Ran P., Han Z. (2026). Microplastics reduce soil detachment capacity by modulating soil physical properties: Experimental evidence from concentration gradients. J. Soils Sediments.

[5] Wang F., Wang Q., Adams C.A., Sun Y.H., Zhang S.W. (2022). Effects of microplastics on soil properties: Current knowledge and future perspectives. J. Hazard. Mater..

[6] Hu J.N., He D.F., Zhang X.T., Li X.Y., Wei G., Zhang Y.L., Ok Y.S., Luo Y.M. (2022). National-scale distribution of micro (meso) plastics in farmland soils across China: Implications for environmental impacts. J. Hazard. Mater..

[7] Xu Z., Zhang L., Jiang G.Y., Ding X.Y., Guo Y.D., Tian Y.Q. (2025). Degradation of mulch films in different soils and its effects on soil properties and ecotoxicology. Environ. Geochem. Health.

[8] Zhang Y., Wang X.T., Li J.Q., Yang Y.T., Gao Y., Wang P.C. (2025). Polyethylene microplastic: Impacts on ryegrass seed germination and seedling development. BMC Plant Biol..

[9] Cai Y.M., Xu Y.Y., Liu G.L., Li B.C., Guo T., Ouyang D., Li M., Liu S., Tan Y.Y., Wu X.D. (2024). Polyethylene microplastic modulates lettuce root exudates and induces oxidative damage under prolonged hydroponic exposure. Sci. Total Environ..

[10] Wang L., Liu Y., Kaur M., Yao Z.S., Chen T.Z., Xu M. (2021). Phytotoxic effects of PE-MPs on the growth of food crops soybean (*Glycine max*) and mung bean (*Vigna radiata*). Int. J. Environ. Res. Public Health.

[11] Sahasa R.G.K., Dhevagi P., Poornima R. (2023). Effect of PE-MPs on seed germination of Blackgram (*Vigna mungo* L.) and Tomato (*Solanum lycopersicum* L.). Environ. Adv..

[12] Aguilar E.G., Albarracín G.J., Uñates M.A., Piola H.D., Camiña J.M., Escudero N.L. (2015). Evaluation of the nutritional quality of the grain protein of new *Amaranthus tricolor* L. varieties. Plant Foods Hum. Nutr..

[13] Asterina Y., Khairunnisa A. (2025). Antioxidant Activity Test of Red Spinach Leaf (*Amaranthus tricolor* L. *us Tricolor* L.) Extract and Fraction with Methode (2, 2-Diphenyl-1 Picrylhydrazyl). SPECTA J. Technol..

[14] Hasyimi M., Iqbal M., Andrifianie F., Triyandi R. (2025). Pharmacological Activities of Red Spinach (*Amaranthus tricolor* L. *us tricolor*). Med. Prof. J. Lampung.

[15] Yu L., Zhang J.D., Liu Y., Chen L.Y., Tao S., Liu W.X. (2021). Distribution characteristics of microplastics in agricultural soils from the largest vegetable production base in China. Sci. Total Environ..

[16] Li Z.Y., Zeng X.L., Sun F.H., Feng T.J., Xu Y.X., Li Z.W., Wu J.H., Wang-Pruski G., Zhang Z.Z. (2023). Physiological analysis and transcriptome profiling reveals the impact of microplastic on melon (*Cucumis melo* L.) seed germination and seedling growth. J. Plant Physiol..

[17] Luo Y., Xiang Y.Z., Ren J. (2025). Effects of chili straw biochar on alfalfa (*Medicago sativa* L.) seed germination and seedling growth on electrolytic manganese residue. Plants.

[18] Liu H.J., Tang H.L., Gao Y.T., An Y.L., Zhou J.F., Li X.Y., Wang S.J., Feng G.Z., Gao Q., Gou Z.C. (2025). The multifaceted mechanisms of microplastic inhibition of tomato plant growth: Oxidative toxicity, metabolic perturbation, and photosynthetic damage. Plant Physiol. Biochem..

[19] Fang Z., Sallach J.B., Hodson M.E. (2024). Size- and concentration-dependent effects of microplastics on soil aggregate formation and properties. J. Hazard. Mater..

[20] Hanif M.N., Aijaz N., Azam K., Akhtar M., Laftah W.A., Babur M., Abbood N.K., Benitez I.B. (2024). Impact of microplastics on soil (physical and chemical) properties, soil biological properties/soil biota, and response of plants to it: A review. Int. J. Environ. Sci. Technol..

[21] Jia X.K., Li H.Q., Tang D.W.S., Xue S., Liu E.K., Yang X.M. (2025). Effects of microplastic contamination on soil nitrogen and its bioavailability in soybean-maize rotation system. Soil Tillage Res..

[22] Singh A., Khare S., Niharika, Gupta P. (2025). Sulfur and phosphorus transporters in plants: Integrating mechanisms for optimized nutrient supply. Plant Physiol. Biochem..

[23] Wang W.X., Xie Y.M., Li H., Dong H.M., Li B., Guo Y.J., Wang Y.T., Guo X.R., Yin T., Liu X.W. (2024). Responses of lettuce (*Lactuca sativa* L.) growth and soil properties to conventional non-biodegradable and new biodegradable microplastics. Environ. Pollut..

[24] Jamil A., Ahmad A., Moeen-ud-din M., Zhang Y.H., Zhao Y.X., Chen X.C., Cui X.Y., Tong Y.D., Liu X.H. (2025). Unveiling the mechanism of micro- and nano plastic phytotoxicity on terrestrial plants: A comprehensive review of omics approaches. Environ. Int..

[25] Guo L.S., Xu X., Wang Q., Park J., Zhou L., Lei H.M., Wang X.H. (2025). Effects of microplastics on the hydraulic properties and pore characteristics of compacted soil. Soil Tillage Res..

[26] Li K., He Q.H., Zhu J., Wang J.Y., Sun C., Tan A., Zhao X.Q., Peng Y.H., Huang C., Cai J.J. (2025). Responses of microbial communities to the addition of different types of microplastics in agricultural soils. Environ. Pollut..

[27] Gao B., Yao H.Y., Li Y.Y., Zhu Y.Z. (2021). Microplastic addition alters the microbial community structure and stimulates soil carbon dioxide emissions in vegetable-growing soil. Environ. Toxicol. Chem..

[28] García-Berumen J.A., Flores de la Torre J.A., Juan A., de Los Santos-Villalobos S., Espinoza-Canales A., Echavarria-Chairez F.G., Gutierrez-Banuelos H. (2025). Phosphorus dynamics and sustainable agriculture: The role of microbial solubilization and innovations in nutrient management. Curr. Res. Microb. Sci..

[29] Xiao C., Zhang W.Y., Liang B.W., Xiong W., Du Y. (2026). An emerging sink for phosphorus in lake ecosystems: Microplastic-enabled iron and phosphorus costabilization in the overlying water. Water Res..

[30] Guo W.J., Ye Z.W., Zhao Y.N., Lu Q.L., Shen B., Zhang X., Zhang W.F., Chen S.C., Li Y. (2024). Effects of different microplastic types on soil physicochemical properties, enzyme activities, and bacterial communities. Ecotoxicol. Environ. Saf..

[31] Li J., Cheng X.Y., Chu G.X., Hu B.W., Tao R. (2023). Continuous cropping of cut chrysanthemum reduces rhizospheric soil bacterial community diversity and co-occurrence network complexity. Appl. Soil Ecol..

[32] Zhao C.R., Yin X.H., Chen J.N., Cao F.B., Abou-Elwafa S.F., Huang M. (2023). Effect of rapeseed straw-derived biochar on soil bacterial community structure at tillering stage of *Oryza Sativa*. Can. J. Microbiol..

[33] Han L.F., Chen L.Y., Feng Y.F., Kuzyakov Y., Chen Q., Zhang S.B., Chao L., Cai Y.P., Ma C.X., Sun K. (2024). Microplastics alter soil structure and microbial community composition. Environ. Int..

[34] Kündgen M., Jogler C., Kallscheuer N. (2025). Substrate utilization and secondary metabolite biosynthesis in the phylum Planctomycetota. Appl. Microbiol. Biotechnol..

[35] López-Mondéjar R., Tláskal V., da Rocha U.N., Baldrian P. (2022). Global distribution of carbohydrate utilization potential in the prokaryotic tree of life. mSystems.

[36] Ye Y., Li X., Xu T., Min W., Guo H.J. (2025). Microbial mechanisms underlying organic phosphorus mineralization and inorganic phosphorus solubilization in arid cotton fields under brackish water drip irrigation and phosphorus fertilization. Ind. Crops Prod..

[37] Huo J.L., Song B.Q., Lin X.C., Riaz M., Zhao X.Y., Liu S.X., She Q.Q. (2024). Ecological characteristics of sugar beet plant and rhizosphere soil in response to high boron stress: A study of the remediation potential. J. Environ. Manag..

[38] Pieters J., Jacobs K., Conradie T.A. (2026). Hidden partners in diversity: Acidobacteriota and their distribution in the cape floristic region. MicrobiologyOpen.

[39] Mcreynolds E., Elshahed M.S., Youssef N.H. (2025). An ecological- evolutionary perspective on the genomic diversity and habitat preferences of the Acidobacteriota. Microb. Genom..

[40] Fuller S., Gautam A. (2016). A procedure for measuring microplastics using pressurized fluid extraction. Environ. Sci. Technol..

[41] Saha A., Baruah P., Handique S. (2024). Assessment of microplastic pollution on soil health and crop responses: Insights from dose-dependent pot experiments. Appl. Soil Ecol..

[42] Lichtenthaler H.K., Wellburn A.R. (1983). Determinations of total carotenoids and chlorophylls a and b of leaf extracts in different solvents. Biochem. Soc. Trans..

[43] Tsikas D. (2017). Assessment of lipid peroxidation by measuring malondialdehyde (MDA) and relatives in biological samples: Analytical and biological challenges. Anal. Biochem..

[44] Strickland T.C., Sollins P. (1987). Improved method for separating light- and heavy-fraction organic material from soil. Soil Sci. Soc. Am. J..

[45] Camargo F.A.O., Gianello C., Vidor C. (1997). Comparative study of five hydrolytic methods in the determination of soil organic nitrogen compounds. Commun. Soil Sci. Plant Anal..

[46] Ge S.F., Zhu Z.L., Ling P., Chen Q., Jiang Y.M. (2018). Soil nutrient status and leaf nutrient diagnosis in the main apple producing regions in China. Hortic. Plant J..

[47] Luo Y., Liu F., Ren J., Zhu J., Luo X.Q., Xiang Y.Z. (2022). Effects of dominant plant growth on the nutrient composition and bacterial community structure of manganese residues. Int. J. Phytoremediation.

